# Autophagy and Non-Alcoholic Fatty Liver Disease

**DOI:** 10.1155/2014/120179

**Published:** 2014-09-10

**Authors:** Vanessa J. Lavallard, Philippe Gual

**Affiliations:** ^1^INSERM, U1065, Bâtiment Universitaire Archimed, Team 8 “Hepatic Complications in Obesity”, 151 Route Saint Antoine de Ginestière, BP 2 3194, 06204 Nice Cedex 03, France; ^2^Faculty of Medicine, University of Nice-Sophia-Antipolis, 06107 Nice Cedex 2, France; ^3^Centre Hospitalier Universitaire of Nice, Digestive Center, 06202 Nice Cedex 3, France

## Abstract

Autophagy, or cellular self-digestion, is a catabolic process that targets cell constituents including damaged organelles, unfolded proteins, and intracellular pathogens to lysosomes for degradation. Autophagy is crucial for development, differentiation, survival, and homeostasis. Important links between the regulation of autophagy and liver complications associated with obesity, non-alcoholic fatty liver disease (NAFLD), have been reported. The spectrum of these hepatic abnormalities extends from isolated steatosis to non-alcoholic steatohepatitis (NASH), steatofibrosis, which sometimes leads to cirrhosis, and hepatocellular carcinoma. NAFLD is one of the three main causes of cirrhosis and increases the risk of liver-related death and hepatocellular carcinoma. The pathophysiological mechanisms of the progression of a normal liver to steatosis and then more severe disease are complex and still unclear. The regulation of the autophagic flux, a dynamic response, and the knowledge of the role of autophagy in specific cells including hepatocytes, hepatic stellate cells, immune cells, and hepatic cancer cells have been extensively studied these last years. This review will provide insight into the current understanding of autophagy and its role in the evolution of the hepatic complications associated with obesity, from steatosis to hepatocellular carcinoma.

## 1. Introduction

**Figure 1:**
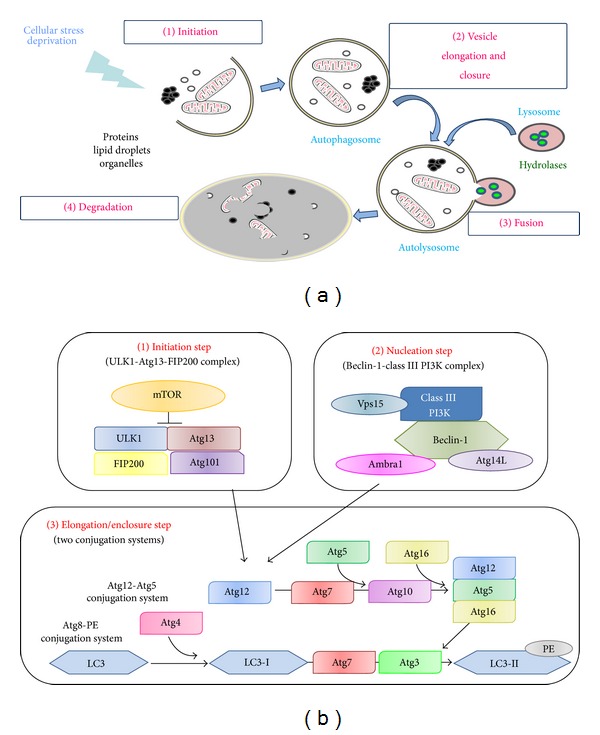
Macroautophagy. (a) Macroautophagy involves the formation of a double-membrane vesicle called the autophagosome. This structure sequesters damaged organelles and misfolded proteins that are degraded by lysosomal enzymes. (b) The* Initiation step* is controlled by the ULK1-Atg13-FIP200 complex, which also contains Atg101. mTOR interacts with and inactivates ULK1 by phosphorylation. Under starvation conditions, mTOR is inactivated leading to ULK1 activation, phosphorylation of Atg13 and FIP200, and consequently the induction of autophagy. The* Nucleation step* requires the Beclin-1-class III PI3K complex including Beclin-1, class III PI3K, Vps15, Atg14L, and Ambra1. The ULK1-Atg13-FIP200 and Beclin-1-class III PI3K complexes recruit two conjugation systems essential for the* elongation and enclosure step*: the Atg12-Atg5 and the Atg8-PE conjugation systems. PE: phosphatidylethanolamine.

Autophagy, or cellular self-digestion, is a catabolic process that targets cell constituents, such as damaged organelles, unfolded proteins, and intracellular pathogens, to lysosomes for degradation [1, 2]. Under basal conditions, autophagy is involved in the degradation of long-lived proteins, whereas the ubiquitin-proteasome pathway, another catabolic process, is responsible for the degradation of short-lived proteins [3, 4]. In response to cellular stress such as nutrient deprivation, an increase in autophagic turnover maintains the cellular energy homeostasis. Three types of autophagy have been identified: macroautophagy, chaperone-mediated autophagy, and microautophagy. Macroautophagy, hereafter referred to as autophagy, involves the formation of a small vesicular sac called the isolation membrane or phagophore. The phagophore encloses a portion of cytoplasm resulting in the formation of a double-membraned structure termed an autophagosome. The autophagosome then fuses with a lysosome leading to the degradation of the cellular constituents sequestered into the autophagosome. Amino acids and other compounds generated by autophagic degradation of macromolecules are released into the cytoplasm for recycling or for energy production (Figure 1(a)) [1]. The origin of the membranes involved in the formation of autophagosomes could be the endoplasmic reticulum (ER), mitochondria, and golgi [5–8]. However, it is still not clear which is/are the major contributor(s).

Microautophagy also involves the sequestration of cellular constituents within lysosomes but in this case through the invagination of the lysosomal membrane. Chaperone-mediated autophagy concerns the sequestration of polypeptides and soluble proteins containing a KFERQ motif in their amino acids sequence. These proteins are bound to a chaperone protein for translocation to lysosomes where binding to the lysosome-associated membrane protein type 2A receptor leads to protein internalization and degradation [9].

In autophagy, the formation of phagophores and autophagosomes requires 18 different autophagy-related proteins, Atg, which were initially identified in yeast [10]. The process of autophagosome formation involves three major steps: initiation, nucleation, and elongation/enclosure (Figure 1(b)). The initiation step is controlled by the ULK1-Atg13-FIP200 complex [11, 12]. The serine/threonine kinase mammalian target of rapamycin, mTOR, a component of the mTORC1 complex, is the main inhibitor of autophagy. The nucleation step requires the Beclin-1-class III phosphatidylinositol 3-Kinase (PI3K) complex that includes Beclin-1, Vps34 (class III PI3K), Vps15, Atg14L/Barkor, and Ambra-1 [13]. The involvement of mTOR and the Beclin-1-class III PI3K complex in the regulation of autophagy are discussed below. Two conjugation systems are involved in the elongation/enclosure step. The first is the conjugation of Atg12 to Atg5 mediated by two ligases Atg7 and Atg10. Atg5 also associates with Atg16 to form the Atg12-Atg5-Atg16 complex. The second involves the cleavage of LC3/Atg8 by Atg4 leading to the soluble form LC3-I, which is then conjugated to phosphatidylethanolamine, PE, via the participation of Atg7 and Atg3. This lipid conjugation forms the autophagic double-membrane-associated LC3-II protein allowing the closure of the autophagic vacuole [14, 15]. LC3-II is used as a marker of autophagosomes [16].

The last phases of autophagic process mediate the autophagy degradation. A large number of factors/actors regulate the autophagosome-lysosome fusion and the lysosomal biogenesis, activation, reformation, and turnover [17]. In the autophagosome-lysosome fusion process, soluble N-ethylmaleimide-sensitive factor attachment protein, cytoskeleton proteins, and small GTPases are involved, for example. This mechanism is sensitive to changes in the membrane lipid composition of autophagosomes and lysosomes that can regulate their fusogenic capacity as it has been reported in lipid-enriched diets [18]. Other important regulator of lysosomal biogenesis, function, and autophagy is the transcription factors EB, TFEB [19, 20]. TFEB coordinates the cellular responses to different stresses, including nutrient starvation, metabolic stress, and lysosomal stress, to maintain cellular homeostasis. Indeed, TFEB regulates the expression of genes involved in lipid metabolism and in the pathways of autophagy and lysosome [19, 20].

Autophagy is a cellular pathway that is crucial for the maintenance of cellular homeostasis, normal mammalian physiology and could play a protective or deleterious role in a range of diseases. Recent studies have reported its role and its regulation in the complications associated with obesity.

## 2. Hepatic Complications Associated with Obesity

**Figure 2:**
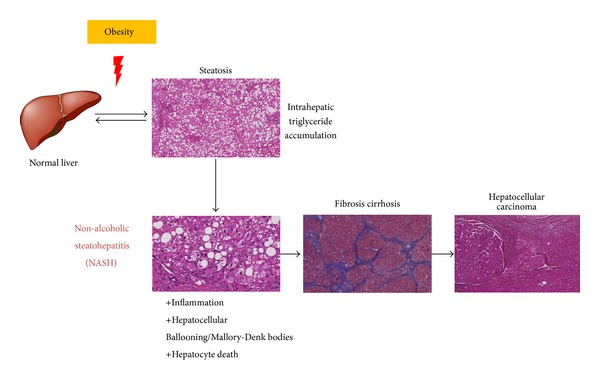
NAFLD. The spectrum of non-alcoholic fatty liver diseases (NAFLD) extends from isolated steatosis (triglyceride accumulation) to steatohepatitis (steatosis with inflammation) (non-alcoholic steatohepatitis [NASH]), steatofibrosis, which sometimes leads to cirrhosis, and hepatocellular carcinoma. Peripheral insulin resistance may represent the “first hit” in the pathogenesis of NAFLD, which leads to hepatic steatosis. Combined hyperglycemia and hyperinsulinemia promote* de novo* lipid synthesis and structural defects in mitochondria within hepatocytes. Moreover, insulin resistance of adipose tissue leads to an enhanced free fatty acid flux to the liver that contributes to steatosis. Steatotic hepatocytes may be vulnerable to a “second hit” induced by cytokines (such as TNF*α*) and oxidative/ER stresses, which lead to the development of steatohepatitis and fibrosis. Apoptotic hepatocytes are engulfed by kupffer cells, which results in their activation and inflammation. The activation of stellate cells by apoptotic bodies or by TGF*β* from activated kupffer cells then leads to liver fibrosis [22].

The incidence of overweight and obesity is rapidly increasing in many Western countries. Obesity leads to numerous adverse metabolic disorders such as dyslipidemia, hypertension, reduced HDL cholesterol, and glucose intolerance. This cluster of metabolic abnormalities is grouped into the so-call metabolic syndrome that increases the risk of cardiovascular diseases, type 2 diabetes and liver complications, the non-alcoholic fatty liver disease (NAFLD). The spectrum of these hepatic abnormalities extends from isolated steatosis with triglyceride accumulation to non-alcoholic steatohepatitis (NASH), steatofibrosis, which sometimes leads to cirrhosis and hepatocellular carcinoma (Figure 2). NAFLD is one of the three main causes of cirrhosis [21]. Despite this major public health concern, apart from lifestyle changes, NAFLD is still difficult to treat as no large study has shown any efficacy of pharmacological treatments for NAFLD.

Insulin resistance is at the core of the pathophysiology of the metabolic syndrome and type 2 diabetes. Insulin resistance is characterized by a decrease in insulin signaling and action. Adipose tissue plays a central role in the control of glucose and lipid metabolism through its ability to control glucose transport, lipid storage, and adipokines secretion. In obesity, the excessive gain of adipose tissue, in particular visceral adipose tissue, causes its dysfunction, which could participate in the development of insulin resistance and other obesity-linked complications such as the NAFLD. Adipose tissue expansion is associated with chronic low-grade inflammation with infiltration of the tissue by immune cells such as dendritic cells, macrophages, and T lymphocytes. Consequently, adipose tissue produces inflammatory cytokines and free fatty acids (FFA) in excess, and adipokine secretion is perturbed. Inflammatory cytokines and FFA antagonize local insulin signaling in adipocytes and also in muscles and liver. In liver, combined hyperglycemia and hyperinsulinemia promote* de novo* lipid synthesis and mitochondrial structural defects within hepatocytes. Moreover, insulin resistance of adipose tissue leads to an enhanced FFA flux to the liver, for example, in contribution to insulin resistance and steatosis. Oxidative and ER stresses play an important role in the alteration of insulin signaling and in the development of liver complications [22]. NASH is characterized by a fatty liver, hepatic inflammation, and substantial death of hepatocytes. Hepatocyte apoptosis is important in the progression of the severity of liver complications. Apoptotic hepatocytes are engulfed by kupffer cells, which results in their activation and inflammation. The activation of stellate cells by apoptotic bodies or by TGF*β* from activated kupffer cells then leads to liver fibrosis [22]. Among the factors involved in this process, upregulation of cell death receptors, such as Fas and the TNF*α* receptor, and of TNF*α* has been reported in the NASH liver. Saturated FFA, sustained ER stress, cytokines, and adipokines could also be involved [22].

Interestingly, it has been recently reported that autophagy regulates food intake, adipose tissue development, hepatic complications, and insulin resistance and plays a protective role against lipotoxicity in *β* cells [23]. Here, we will provide insight into the current understanding of the role of autophagy in the liver complications associated with obesity.

## 3. Hepatic Autophagy in Obesity

**Figure 3:**
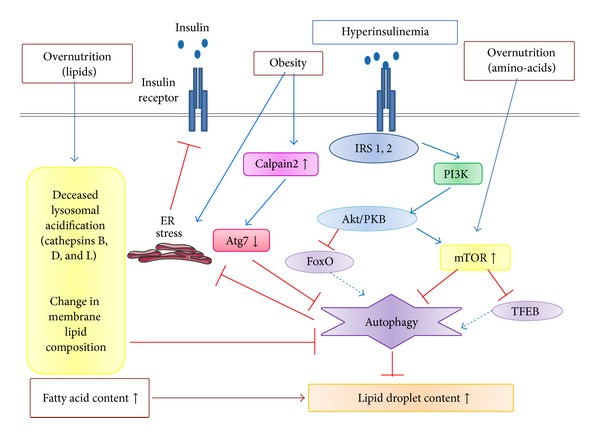
Molecular mechanisms of the impairment of hepatic autophagy in obesity. Short-term inhibition can be produced through the mTOR complex. Long-term regulation could occur* via* the transcription factors FoxO and TFEB, which control the transcription of autophagic genes and are inhibited by insulin-induced activation of Akt/PKB and mTOR, respectively. mTOR could be overactivated in the liver, presumably as a result of an increased amino acid concentration following overnutrition and/or hyperinsulinemia. An obesity-induced increase in the calcium-dependent protease calpain-2 could also lead to the degradation of Atg7 and then to defective autophagy. A defect in lysosomal acidification and a reduction in cathepsins B, D, and L expression, which impaired substrate degradation in autolysosomes, have also been reported. Finally, a defect in fusion in organelles including autophagosome-lysosome fusion attributed to HFD-induced changes in the membrane lipid composition. A defect in hepatic autophagy and the associated decrease in the rate of lysosomal degradation contribute to a further increase in the ER stress induced by nutrient overload and insulin resistance.

Under physiological conditions, autophagy participates in the basal turnover of lipids by engulfing and degrading lipid droplets. Autophagy is inhibited by the insulin-, amino acid-mTOR signaling pathway* via* both short- and long-term mechanisms of regulation. Short-term inhibition can be produced by the mTOR complex. Long-term regulation occurs* via* the transcription factors FoxO and TFEB, which control the transcription of autophagic genes and are inhibited by insulin-induced activation of Akt/PKB and mTOR, respectively [23]. In obesity, the level of autophagy could be decreased in hepatocytes. Several mechanisms may account for this decline (Figure 3).

- An obesity-induced increase in the calcium-dependent protease calpain-2 leads to the degradation of Atg7 and then to a defective autophagy. Acute inhibition of calpain is able to restore Atg7 expression [24]. How obesity enhances the activity or expression of the hepatic calpain 2 has not yet been elucidated.
- In obese mice with hepatic steatosis, the autophagy inhibitor mTOR is overactivated in the liver, presumably as a result of an increased amino acid concentration following overnutrition. Indeed, it has been previously shown that the overactivation of mTOR by infusion of an amino acid mixture can result in liver and muscle insulin resistance because of phosphorylation and inhibition of IRS1 by S6 kinase, a downstream target of mTOR [25, 26].
- Although controversial, hyperinsulinemia may also contribute to downregulation of autophagy in obese mice. Indeed, Akt/PKB, a key molecule in the insulin pathway, decreases autophagy in the liver of obese mice [27]. However, destruction of insulin production by *β* cells with streptozotocin does not increase autophagy in the liver of obese mice [24], in contrast to lean mice [27]. The reasons for these discrepancies are unclear.
- A defect in lysosomal acidification and a reduction in cathepsin L that impaired substrate degradation in autolysosomes have also been reported for obese ob/ob mice. This is associated with an increased autophagosome number and normal fusion of autophagosomes to lysosomes [28]. The same team recently reported that cathepsin B, D, and L expression was significantly decreased in the liver from NAFLD patients [29].
- Defective autophagosome-lysosome fusion has also been reported in livers from high fat diet- (HFD-) induced obese mice. This defect was attributed to HFD-induced changes in the membrane lipid composition [18]. A defect in hepatic autophagy and its associated decrease in the rate of lysosomal degradation contribute to a further increase in the ER stress. This could be induced by nutrient overload in an inflammatory milieu [24, 30]. Together, a decline in autophagy and an increase in ER stress lead to insulin resistance [24].

## 4. Hepatic Steatosis

In response to a moderate increase in lipid availability or during nutrient deprivation, hepatic autophagy degrades lipid droplets to provide FFA for ATP production. In contrast, a sustained availability of lipids, induced by a long-lasting HFD challenge, inhibits hepatic autophagic turnover [31]. This ability of autophagy to degrade lipid droplets in hepatocytes has been termed lipophagy. Singh et al. reported for the first time that autophagy regulates lipid metabolism by eliminating triglycerides and by preventing development of steatosis. Inhibition of macroautophagy by genetic knockdown of the autophagy gene* atg5*, or pharmacological inhibition with 3-methyladenine in cultured hepatocytes challenged with a lipid load, significantly increased the cellular triglyceride content. Excessive triglycerides and cholesterol were retained in lipid droplets because of a decreased rate of lipolysis and the resultant reduction in fatty acid *β*-oxidation in cells in which macroautophagy was inhibited. Lipid movement through the autophagic pathway was confirmed by fluorescence microscopy demonstrating colocalization of lipid with autophagosomes and lysosomes, electron microscopic evidence of lipid in the autophagic vacuoles, and immunogold staining demonstrated an association of the autophagosome-associated protein microtubule-associated LC3 protein with lipid droplets. The LC3 protein directly interacts with lipid droplets before autophagosome formation. Lysosomes merge only with autophagosome-associated lipid droplets [31]. These results have been confirmed [32].

Differences in the activation of autophagy, p53, damage-regulated autophagy modulator (DRAM), and BAX expression have also been observed in function to the severity of the hepatic steatosis. In the mouse model of mild (20 weeks of HFD) and severe hepatosteatosis (40 weeks of HFD), p53 expression increased in both mild and severe hepatic steatosis, and increased DRAM expression and autophagy were identified in mild hepatosteatosis, whereas higher BAX expression was observed in severe hepatosteatosis [33]. From* in vitro* approaches, the authors proposed that mild steatosis induced autophagy and apoptosis mostly via a p53/DRAM pathway. In severe steatosis, apoptosis was mainly dependent on p53-induced expression of BAX, which also localized to mitochondria [33]. Since discrepancies between* in vivo* and* in vitro* approaches exist, future investigations are necessary to confirm this potential mechanism.

HFD-fed mice (16 weeks) show impairment in the hepatic autophagic function, as demonstrated by the decreased mobilization of lipid into the autophagic compartment [31]. Lipid accumulation altered the membrane structure, and a resultant decrease in the efficiency of fusion between autophagosomes and lysosomes may explain the inhibitory effect on macroautophagy of lipid accumulation induced by a HFD. However, in livers with a deficiency in autophagy, an alternative protective mechanism could then take place. Indeed, Kim et al. have recently reported that mice with a deficiency in hepatic autophagy displayed induction of fibroblast growth factor 21 (FGF21), resistance to diet-induced obesity, and amelioration in insulin resistance. The deficiency in autophagy and subsequent mitochondrial dysfunction could promote FGF21 expression, which in turn protects from diet-induced obesity and insulin resistance [34]. In NAFLD patients, the serum level of FGF21 is also modified. In 146 overweight patients, it was reported that the serum levels of FGF21 were significantly higher in NASH patients. FGF21 also correlated positively with the triglyceride level, metabolic syndrome, steatosis grade, lobular inflammation, and fibrosis [35]. Interestingly, the effects of LY2405319 (LY), an engineered FGF21 variant [36, 37], in a randomized, placebo-controlled, double-blind proof-of-concept trial on 46 patients with obesity and type 2 diabetes has been recently reported. Patients received placebo or 3, 10, or 20 mg of LY daily for 28 days. LY treatment produced a significant improvement in dyslipidemia. Favorable effects on body weight, fasting insulin, and adiponectin were also detected. While only a trend toward glucose lowering was observed, FGF21-based therapies may be effective for the treatment of selected metabolic disorders [38]. Additional exploration would be necessary in order to assess the full range of LY effects and the potential to achieve significant antidiabetic efficacy. Further, the effect of LY administration on liver complications (hepatic steatosis) should be evaluated. The improvement of insulin sensitivity by LY could reduce the free fatty acid flux to the liver that contributes to steatosis.

## 5. Activation of Hepatic Autophagy Decreases Liver Steatosis

Activation of autophagy in hepatocytes could constitute a therapeutic approach against hepatic complications. To illustrate this, it has been reported that hepatic overexpression of Atg7 in HFD-fed mice or ob/ob mice improved the condition of the fatty liver and insulin resistance [24].

Starvation induces hepatic autophagy and increases delivery to the liver of FFA from adipose tissue. The liver of starved mice displayed an increase in the number of lipid droplets, autophagosomes, lysosomes, and autophagolysosomes [31]. Hepatocyte-specific Atg7-deficient mice are characterized by hepatomegaly and accumulation of poly-ubiquitinylated proteins, as previously reported by Komatsu et al. [39]. Hepatic triglycerides and the cholesterol content are also increased in these mice, which confirm the crucial role of autophagy in the regulation of lipid storage. The activation of autophagy by starvation is a complex mechanism. A new actor has been identified: acetyl-coenzyme A (AcCoA). AcCoA is a major integrator of the nutritional status at the crossroads of fat, sugar, and protein catabolism and cytosolic AcCoA functions as a central metabolic regulator of autophagy. Nutrient starvation causes rapid depletion of AcCoA and induction of autophagy* via* the reduction in the activity of acetyltransferase EP300, a suppressor of autophagy, by high AcCoA levels [40].

Enhancers of autophagy such as carbamazepine and rapamycin have been recently tested in HFD-obese mice and both had protective effects in reducing steatosis and in improving insulin sensitivity. The agents were given two or three times in the last week of a 12-week feeding scheme. This short-term treatment could significantly reduce hepatic steatosis and hepatic and blood triglyceride levels. The plasma ALT level was also noticeably, although not statistically significantly, reduced. Interestingly, insulin resistance was improved as evaluated by the level of blood glucose and insulin [41]. While rapamycin and carbamazepine are already approved for other human clinical uses, the lack of specificity and the absence of organ or cell selectivity are the major limitations of these compounds.

The beneficial effects of coffee on hepatic steatosis and the link with autophagic flux have been recently tested. The recent evidence of the beneficial effects of coffee on the liver came from epidemiologic studies that revealed a strong association of drinking coffee with decreased serum hepatic enzymes, including GGT, AST, and ALT, in persons with a high risk of liver injury, such as in alcoholic, diabetic, and in viral infections [42]. Recent epidemiologic studies further support the finding that drinking coffee reduces the risk for fatty liver, fibrosis, and hepatocellular carcinoma in NAFLD patients [43, 44]. Sinha et al. recently reported that mice given a HFD for 4 weeks, then continued HFD with 0.05% (w/v) caffeine in the drinking water for the next 4 weeks displayed an induction of hepatic autophagy (lipophagy) with a decrease in hepatic steatosis [45]. This treatment was nevertheless associated with a decrease in HFD-induced obesity. Using genetic, pharmacological, and metabolomic approaches on hepatic cells and on the liver, the authors showed that caffeine induced lipophagy and mitochondria *β*-oxidation leading to a reduction in the intra-hepatic lipid content. Caffeine may inhibit PI3K-AKT and, in turn, inhibit mTOR to trigger autophagy by activating the ULK1 complex. The later includes ULK1, Atg13, FIP200, and Atg101. Autophagy selectively removes excess lipid droplets to generate FFA. Decreased mTOR levels induced TFEB nuclear translocation by decreasing TFEB phosphorylation. TFEB upregulates expression of autophagy and lysosomal genes, as well as PGC-1*α* and PPAR*α*, which burn FFAs by increasing mitochondria *β*-oxidation. Thus, caffeine protects against fatty liver by coordinating the induction of lipophagy and mitochondrial *β*-oxidation [45, 46].

## 6. Hepatic Insulin Resistance

Recent studies have suggested that ER stress could be the link between obesity, insulin resistance, and type 2 diabetes [47–49]. The inhibition of hepatic ER stress reduced liver steatosis [50]. Defective hepatic autophagy in obesity could promote ER stress and cause insulin resistance [47]. Since autophagy is known to eliminate mis-/unfolded proteins, and impairment in hepatic autophagy could lead to accumulation of mis-/unfolded proteins and induction of ER stress. In the liver of obese mice, it has been reported that a decrease in autophagy promotes ER stress leading to insulin resistance [24, 51]. Overexpression of Atg7 in the liver of obese mice significantly reduced ER stress, decreased the triglyceride content, and improved glucose tolerance and insulin sensitivity [24]. Thus, a vicious cycle takes place: hyper-insulinemia negatively regulates hepatic autophagy in the steatotic liver and the decline in hepatic autophagy enhances ER stress and insulin resistance (Figure 3).

## 7. Steatohepatitis (NASH)

The possible contribution of autophagy to the evolution of steatosis to NASH has not yet been fully elucidated [52]. The diagnosis of NASH still requires a liver biopsy and is defined by histological features including steatosis, lobular inflammation, and hepatocellular ballooning [53, 54]. Evaluating hepatic fibrosis using a histopathological algorithm and scoring systems to diagnosis NASH is still under debate [55]. Hepatic cell injury including hepatocyte death and hepatocellular ballooning are key features of NASH. Inhibition of autophagy and the accumulation of p62 could be involved in the formation of the Mallory-Denk bodies (MDB). MDB are mainly found in ballooned hepatocytes, one of the components used for NASH diagnosis and for the scoring system [53, 54]. In contrast, activation of autophagy by rapamycin leads to MDB resolution in mice [56].

A recent report has evaluated autophagic markers in obese patients with hepatic steatosis (*n* = 26), patients with steatosis with NASH, and fibrosis (*n* = 23) versus normal liver (*n* = 34). A significant increase in the LC3-II/LC3-I ratio was detected in both patients with steatosis and NASH compared with subjects with a normal liver. A progressive increase in the amount of p62 was observed in obese patients with steatosis and in obese patients with NASH compared to control patients. The accumulation of p62 and the LC3II/LC3I ratio could indicate that autophagy was decreased in both patients with hepatic steatosis and NASH [57]. The level of autophagic markers in patients with the same grade of steatosis and fibrosis with or without NASH remains to be investigated, to determine if a discrepancy exists between steatosis and NASH. In contrast, NASH patients had a significant increase in hepatic ER stress (ATF4 at the mRNA level; CHOP and GRP78 at the mRNA and protein levels) compared with patients with steatosis [57]. In a mouse model of NASH (methionine/choline-deficient diet), the authors also demonstrated that the autophagic flux was impaired in the liver [57]. We would like to underline the fact that the evaluation of autophagic flux* in vivo* in mice is still difficult and even more difficult in human samples. Further, assessing autophagy by the LC3-II/LC3-I ratio and p62 level need always caution. The levels of LC3-I can also vary. In addition, p62 levels are enhanced by other factors such as stress, or proteasome inhibition, which are mostly independent of changes in autophagy.

The immune system plays an important role in the evolution of NAFLD [22]. For example, the dysregulation of the balance in M1 (pro-inflammatory) versus M2 (anti-inflammatory) macrophages is emerging as a central mechanism governing the pathogenesis of NAFLD [58, 59]. In the last few years, the regulation of the immune system by autophagy has been reported [60–63]. The evaluation of the autophagic flux and its role in specific cells such as macrophages, T cells, and neutrophils in the NAFLD liver remains to be fully investigated. To illustrate this, the role of autophagy in tumor-associated macrophages in hepatocellular carcinoma is discussed below.

## 8. Hepatic Fibrosis

Hepatocyte apoptosis and inflammation are key players in the progression of the severity of liver complications. With the aim of simplifying hepatic fibrogenesis [64], hepatic stellate cells (HSCs) in response to damage (hepatocyte death…) and inflammation (TGF*β*) differentiate into myofibroblast-like cells, which produce most extracellular matrix components. Prevention of hepatocyte death can prevent fibrosis. For example, pan-caspase inhibitors reduced hepatic fibrosis in db/db mice feed with a methionine/choline-deficient diet [65]. Circulating levels of markers of hepatocyte death and/or apoptosis also increased with the severity of hepatic fibrosis in obese or alcoholic patients [66, 67].

**Figure 4:**
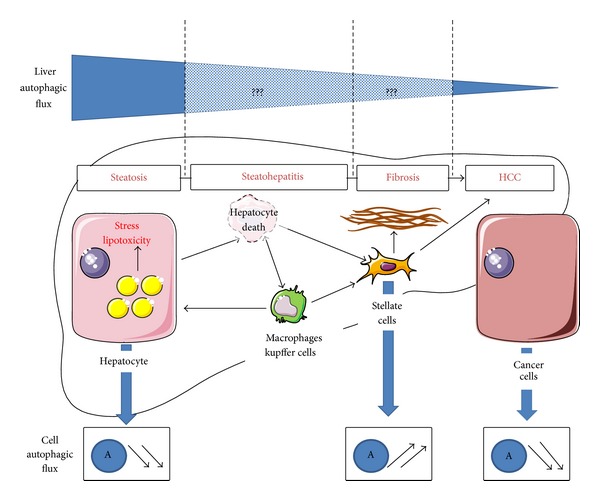
Autophagy and NAFLD. The evolution of NAFLD could be associated with dynamic regulation of autophagy. In the steatotic liver and hepatocytes, the autophagic flux is decreased and is associated with an alteration in the metabolic homeostasis and vulnerability of the liver. The low number of reports concerning the NASH liver does not allow one to clearly establish if an additional alteration in the autophagic flux occurs with inflammation. In the fibrotic liver, activation of autophagy in HCSs regulates their activation. In HCC, a decrease in autophagy in cancer cells and tumor-associated macrophages (M2 phenotype) facilitate tumor initiation and progression, respectively. Furthermore, liver complications increase with age and it is well established that hepatic autophagy is impaired in aging. It is thus difficult to obtain a clear picture of the level of autophagy in liver diseases in reinforcing the notion of dynamic processes. Additional studies are required to better understand the role of autophagy in the liver and in specific liver cells in NAFLD. However, it is obvious that important deregulation of hepatic autophagy facilitates the development of NAFLD. (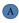: autophagy.)

Since autophagy plays a hepatoprotective role, the steatotic liver with decreased hepatocyte autophagy is more vulnerable to injury induced by inflammation (TNF*α*, FASL) and stresses (ER and oxidative) and is therefore more prompted to develop fibrosis. Furthermore, activation of autophagy in HSCs has recently emerged as an additional mechanism involved in their activation (Figure 4) [64, 68, 69]. The increase in autophagic flux in activated HSCs leads to the loss of perinuclear lipid droplets (containing retinyl esters) associated with the transdifferentiation of quiescent HSC towards the activated phenotype [68, 69]. It has been hypothesized that the metabolism of lipid droplets mainly due to autophagy (probably by lipophagy) may provide the cellular energy that is critical for fueling catabolic pathways of HSCs activation. Under conditions of stress, ER stress (IRE1*α*-Xbp1-p38 pathway) could play an important role in the activation of autophagy and in turn fibrogenic activity of HSCs [70]. In a mouse model of liver fibrosis (carbon tetrachloride or thioacetamide), hepatic autophagy is activated and the loss of autophagic function in HSCs (Atg7^F/F^—glial fibrillary acidic protein—Cre mice) reduced their activation, fibrogenesis, and matrix accumulation. This inhibition of autophagy in HSCs also prevented the loss of perinuclear lipid droplets* in vivo* [68]. Unfortunately, this has not yet been evaluated in a mouse model of NAFLD.

Thus, in addition to selective activation of autophagy in hepatocytes to decrease cell death, selective reduction of autophagic activity in fibrogenic cells in the liver unveils a potential new therapeutic strategy for liver fibrosis. It seems thus important to well targeting the liver cells and disease (severe fibrosis versus steatosis/NASH) for the treatment. Activation of autophagy in hepatocyte in early stages of NAFLD (steatosis/NASH) could prevent their progression to fibrosis. Indeed, limiting liver injury could be a therapeutic way to prevent the progression of hepatic complications [71]. It has been reported that a pan-caspase inhibitor or overexpression of the anti-apoptotic Bcl2 protein reduced fibrosis in an animal model of NAFLD and fibrosis, respectively [65, 72]. Furthermore, two years of treatment with ursodeoxycholic in combination with vitamin E improved the level of hepatic enzymes AST/ALT and hepatic steatosis of patients with NASH [73]. Ursodeoxycholic is commonly used in the treatment of cholestatic liver disorders but its potential ability to prevent hepatocyte apoptosis has been evaluated in NAFLD [74]. Larger trials are warranted.

## 9. Hepatocellular Carcinoma, HCC

Autophagy is also involved in hepatocarcinogenesis, while its role remains controversial. Autophagy could play a dual role in cancer initiation and in cancer survival. First, autophagy eliminates senescent and injured cells, thereby limiting chromosomal instability and suppresses tumor initiation. Second, autophagy could provide energy by recycling damaged organelles, DNA, aggregated proteins, and pathogens to maintain energy balance, which promotes cancer cell survival [75]. In addition, the regulation of autophagy in liver macrophages and more specifically in tumor-associated macrophages could also play an important role in the development of HCC.

To illustrate the tumor suppressor role of autophagy, it has been reported that mice with heterozygous disruption of beclin-1 have a high frequency of spontaneous hepatocellular carcinoma [76]. Similarly, the deletion of Atg5 or Atg7 in liver resulted in the increasing incidence of benign liver adenomas [77]. Further, the expression of several autophagy related genes (*atg5*,* Atg7,* and* Atg6*/*beclin-1*) and their corresponding autophagic activity is decreased in HCC cell lines compared to normal hepatic cell lines [77, 78]. Similarly, beclin-1 mRNA and protein levels are lower in HCC tissue samples than in adjacent nontumor tissues from the same patients [78]. In 300 patients with HCC, the expression of Beclin-1 correlated with disease-free survival and overall survival only in the Bcl-xL^+^ patients. Multivariate analyses revealed that Beclin-1 expression was an independent predictor for disease-free survival and overall survival in Bcl-xL^+^ patients. Further, Beclin-1 expression correlated with tumor differentiation in Bcl-xL^+^ but not in Bcl-xL^−^ HCC patients. These data suggest that a defect in autophagy synergizes with altered apoptotic activity and facilitates tumor progression and poor prognosis of HCC [78].

Since autophagy negatively regulates stresses and prevents cell death, its activation could also be involved in the survival of cancer. Indeed, LC3 was highly expressed in HCC compared with noncancerous tissues and correlated with tumor size. In addition, LC3 was an independent predictor of HCC recurrence after surgery only in the context of large tumors [79]. Furthermore, autophagy induced by oncogenic K-Ras mediates functional loss of mitochondria during cell transformation to overcome an energy deficit resulting from glucose deficiency [80].

In addition, autophagy could also act* via* the regulation of the function of tumor-associated macrophages. It is well established that the tumor-associated macrophage density in human cancer correlates with poor prognosis in most human cancers and the inhibition or enhancement of the macrophage density in tumors by genetic and pharmacological approaches, respectively, inhibits or promotes tumor angiogenesis, growth, and progression [62, 81, 82]. Further, the polarization of macrophages into the M2 (anti-inflammatory) phenotype favors tumor progression, while M1 (proinflammatory) macrophages exert an anticancer activity. Tumor-associated macrophages could sense factors from the tumor microenvironment that lead to their polarization. Autophagy is involved in this process [62, 81]. For example, the TLR2 deficiency causes a reduction in macrophage infiltration but also induces significant suppression of autophagy associated with a decrease in the hepatic expression of tumor necrosis factor, interferon gamma, and [C-X-C motif] ligand 2. This enrichment in M2 macrophages in turn promotes hepatocarcinogenesis [83].

A specific enhancement in autophagy in tumor-associated macrophages could enhance polarization into the M1 phenotype and could have a beneficial effect. The activation of the mTOR-TSC2 pathway, a key negative regulator of autophagy, is critical for macrophage polarization toward the M2 phenotype to promote tumor angiogenesis and growth in mouse hepatocellular carcinoma models. In contrast, inhibition of this pathway by for example rapamycin/silorimus exerts the opposite effects [84]. Interestingly, the use of rapamycin/silorimus as an immunosuppressor displays beneficial responses in patients. Indeed, survival after liver transplantation has been evaluated according to the immunosuppression protocol applied to 2491 adult recipients of isolated liver transplantation for HCC and 12,167 for non-HCC. These patients remained on stable maintenance of immunosuppression protocols for at least 6 months after transplant. Treatment with rapamycin was associated with improved survival after transplantation for HCC. Interestingly, rapamycin showed a trend toward lower rates of survival in non-HCC recipients, confirming the specificity of its beneficial impact for cancer patients [85].

Activation of autophagy in HCC targeting tumor-associated macrophages may represent a promising and effective strategy for liver cancer therapy. In contrast, the inhibition of autophagy in malignant cells may be a novel strategy to improve the efficacy of anticancer therapy. However, further investigations are required to determine the role of autophagy as a function of the type of tumor, stage, and genetic context.

## 10. Aging

The prevalence of type 2 diabetes and liver complications increases with age and it is well established that autophagy and chaperone-mediated autophagy are impaired in aging [86, 87]. This decline is associated with an increase in the lipid content in different organs such as the liver. As seen above, excessive accumulation of lipids alters further autophagic turnover and its protective role against accumulation of lipid droplets and insulin resistance. A link between aging, loss of mitochondrial function, and ROS production has also been reported [86, 88]. Calorie restriction enhances longevity and this may be due, at least in part, to activation of autophagy, in particular lipophagy and mitophagy leading to a decrease in the lipid content and in oxidative stress [89–91]. Aging, dysfunctional mitochondria, and oxidative stress contribute to the development of type 2 diabetes and liver complications [92, 93]. The increase in autophagic turnover could enhance mitochondrial turnover and biogenesis, and, consequently, decrease ROS production. This may contribute to life span extension and prevent or delay the development of complications associated with obesity [94]. The development of NAFLD has recently been examined in mice of different ages (2, 8, and 18 months) in response to a fixed period 16 weeks of HFD. Weight gain, insulin resistance, and hepatic steatosis were equivalent for the three ages. In contrast, liver injury occurred exclusively in the two older ages and older mice had an elevated innate immune response with more M1 (proinflammatory) macrophages. Aged hepatocytes were further selectively sensitized to the Fas death pathway* in vitro*. Aging thus leads to increased hepatocellular injury and inflammation upon HFD challenge [95]. Unfortunately, the level of autophagy was not evaluated in this study.

## 11. Conclusions

Autophagy is a crucial physiological process in providing nutrients to maintain vital cellular functions during fasting, but also to purge the cell of superfluous or damaged organelles, lipids, and misfolded proteins in obesity. Obesity is a complex multifactorial chronic disease affecting multiorgans and physiological responses [22]. It is clear that modifications in the autophagic turnover during obesity mediate protective or deleterious responses depending on the cell/organ [23]. This review summarized the knowledge of the role of autophagy in specific cells, including hepatocytes, macrophages, HSCs and cancer cells, and liver diseases from steatosis to HCC in the context of obesity (Figure 4). Some drugs that modulate autophagy are already approved for other human clinical: rapamycin, carbamazepine, cisplatin, and metformin promote autophagy in some cells, while the antimalaria compound chloroquine (which increases the intralysosomal pH) inhibits autophagic turnover. However, the lack of specificity and the absence of organ or cell selectivity are the major limitations of these compounds. Better understanding of the molecular mechanisms of autophagy and its implication in specific liver cells, such as hepatocyte, HSCs, macrophages but also endothelial and other immune cells, during the evolution of the liver complications will highlight new potential therapeutic targets.

## Abbreviations

AcCoA: Acetyl-coenzyme A
Akt/PKB: Protein kinase B
ALT: Alanine transaminase
AST: Aspartate transaminase
ATF4: Activating transcription factor 4
Atg: Autophagy-related protein
BAX: Bcl-2-associated X protein
Bcl-2: B-cell lymphoma 2
Bcl-xL: B-cell lymphoma extra-large
CHOP: C/EBP-homologous protein
DRAM: Damage-regulated autophagy modulator
ER: Endoplasmic reticulum
FASL: Fas ligand
FFA: Free fatty acids
FGF21: Fibroblast growth factor 21
FIP200: Focal adhesion kinase family interacting protein of 200 kD
FoxO: Forkhead box protein O
GGT: Gamma-glutamyl transferase
GRP78: Glucose regulate protein 78
HCC: Hepatocellular carcinoma
HDL: High density lipoprotein
HFD: High fat diet
HSCs: Hepatic stellate cells
IFN: Interferon
IRE1: Inositol requiring enzyme 1
LAMP-2A: Lysosome-associated membrane protein type 2A
LC3: Microtubule-associated protein 1A/1B-light chain 3
MDB: Mallory-Denk Bodies
mTOR: Mammalian target of rapamycin
NAFLD: Nonalcoholic fatty liver disease
NASH: Nonalcoholic steatohepatitis
OA: Oleic acid
PA: Palmitic acid
PGC-1: Peroxisome proliferator-activated receptor gamma coactivator 1
PI3K: Phospho-inositide 3-kinase
PPAR: Peroxisome proliferator-activated receptor
TFEB: Transcription factor EB
TGF: Transforming growth factor
TLR: Toll-like receptor
TNF: Tumor necrosis factor
Xbp1: X-box binding protein 1.
